# A Fast and Reliable Method for Simultaneous Waveform, Amplitude and Latency Estimation of Single-Trial EEG/MEG Data

**DOI:** 10.1371/journal.pone.0038292

**Published:** 2012-06-25

**Authors:** Wouter D. Weeda, Raoul P. P. P. Grasman, Lourens J. Waldorp, Maria C. van de Laar, Maurits W. van der Molen, Hilde M. Huizenga

**Affiliations:** Department of Psychology, University of Amsterdam, Amsterdam, The Netherlands; Cuban Neuroscience Center, Cuba

## Abstract

The amplitude and latency of single-trial EEG/MEG signals may provide valuable information concerning human brain functioning. In this article we propose a new method to reliably estimate single-trial amplitude and latency of EEG/MEG signals. The advantages of the method are fourfold. First, no a-priori specified template function is required. Second, the method allows for multiple signals that may vary independently in amplitude and/or latency. Third, the method is less sensitive to noise as it models data with a parsimonious set of basis functions. Finally, the method is very fast since it is based on an iterative linear least squares algorithm. A simulation study shows that the method yields reliable estimates under different levels of latency variation and signal-to-noise ratioÕs. Furthermore, it shows that the existence of multiple signals can be correctly determined. An application to empirical data from a choice reaction time study indicates that the method describes these data accurately.

## Introduction

Single-trial amplitude and latency of EEG/MEG signals may contain valuable information concerning human brain functioning. Amplitude and latency of signals may change during the course of an experiment, for example due to learning or habituation. In addition, particular groups of subjects may be characterized by increased amplitude or latency variation. For example, increased latency variation may be associated with ADHD [1], ageing [2], [3], and low intelligence scores [4].

Studying inter-trial differences requires that estimates of single-trial amplitude and latency are accurate and reliable. This may be a daunting task given the complexity of EEG/MEG data: single-trial EEG/MEG data have a low signal-to-noise ratio (SNR, [2]), and are usually composed of signals from multiple brain processes [5].

Several methods have been proposed to derive single-trial amplitudes and latencies. These methods differ in several ways. First, some methods require an a-priori template function, whereas other methods do not. That is, some methods require that the shape of the signal of interest is defined before analysis (for example, [6], [7]). Second, some methods only allow for either amplitude or latency variation (for example, [8]), whereas other incorporate both types of variation (for example, [9]). Third, some methods assume that the data consist of one underlying signal (for example, [8], [9]) whereas others allow multiple signals each with their own amplitude and latency variation (for example, [7]). The latter is certainly an advantage since it might very well be the case that some early signals do not show marked inter-trial variability whereas some later signals do show variability. Fourth, some methods are susceptible to noise (cf. [10]), whereas in others this susceptibility is reduced by incorporating basis functions. The purpose of the present paper is to combine the strengths of all these methods into one framework, Single-trial Waveform, Amplitude and Latency Estimation (SWALE). First however, we review existing methods in more detail.

A common, and simple, approach to obtain single-trial estimates is peak-picking. Peak-picking entails smoothing of single-trial data with a low-pass filter and searching for the signal maximum within a specified time window to determine amplitude and latency in each trial [11]. Advantages are that no template has to be defined, and that both amplitude and latency can be estimated. However, it is not possible to test whether multiple signals are present. Furthermore the method is very susceptible to noise [10].

A different approach is to explicitly model the signal in each single-trial. Pham et al. [8] assume that an EEG/MEG trial can be modeled by a waveform with trial specific latency. Parameters of the waveform and trial specific latencies are estimated in the frequency domain. This method was extended by Jaskowski et al. [9] to also allow estimation of trial specific amplitudes. Major advantages are that no template is required, since the waveform is estimated, and that the method incorporates both trial varying amplitudes and latencies. Disadvantages are that the method does not allow for multiple signals and that the method does not perform optimally in low SNR conditions [10].

**Figure 1:**
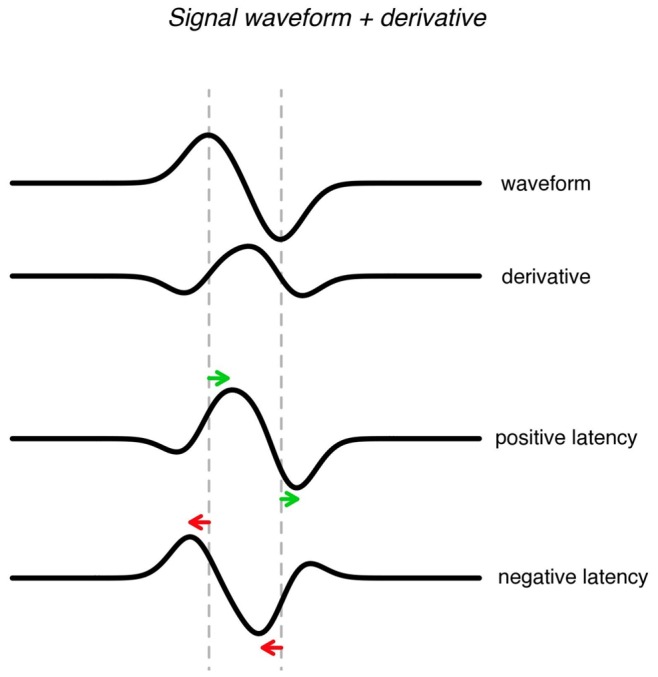
Modeling latency using waveform and its first-order derivative.

A different modeling approach is based on a technique that is also used in the analysis of fMRI data. In fMRI analysis the haemodynamic response (i.e. the response of the brain to a stimulus) is often modeled by a waveform plus its first order derivative (see Figure 1) to allow for differences in latency [12].

Mayhew et al. [7] used this method to estimate single-trial EEG amplitude and latency in a multiple linear regression framework. By regressing the data to a-priori specified template functions and their derivatives, estimates of single-trial amplitude and latency are obtained. The method has the advantage that it can be used to estimate single-trial amplitude and latency of multiple signals. However, it requires that a template is specified for each signal. Mayhew et al. [7] use the averaged data as a template, however this template might be biased in the presence of large latency variation [6], [13]. Another disadvantage is that the template requires as many parameters as there are timepoints, and therefore is very susceptible to noise.

In order to arrive at reliable estimates of single-trial amplitudes and latencies, we combine the aforementioned methods into the SWALE framework. More specifically, we extend the approach of Mayhew et al. [7] such that no a-priori template is required and such that noise sensitivity is diminished. Instead of a template we estimate waveforms from the data (cf. [9]) and model these waveforms with a parsimonious set of basis functions that reduces noise sensitivity. The framework can be extended to model data with multiple waveforms and it can explicitly test for the necessary number of waveforms. Furthermore, estimation of parameters is embedded in an iteratively least squares framework [14] and is therefore very fast. The SWALE framework is available as open source software and can be downloaded from the corresponding author’s website http://home.medewerker.uva.nl/w.d.weeda1/.

In the following we first explain the method in more detail: we formulate the model, outline parameter estimation, and indicate how the optimal number of basis functions and the optimal number of waveforms can be obtained by means of statistical model selection. Second, we report a simulation study on the characteristics of the method. Third, we illustrate the method with an analysis of empirical data obtained in a choice reaction time (CRT) experiment. Finally, we discuss advantages and limitations and provide some extensions.

## Methods

**Figure 2:**
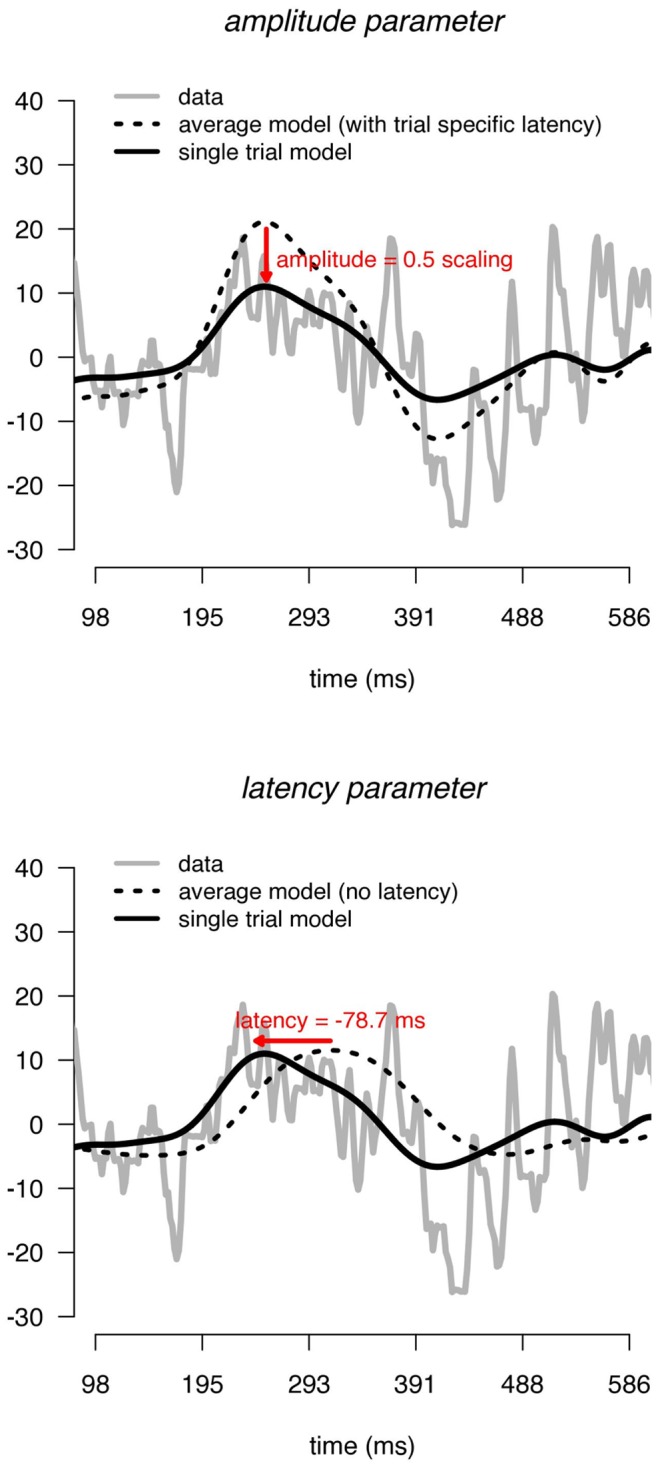
Effect of amplitude and latency parameters on the single-trial model. Solid black lines indicate the single-trial model. Dashed lines indicate the average model. Light grey lines indicate the data.

The rationale of the SWALE framework is to model single EEG/MEG trials by the sum of (i) an overall waveform plus (ii) its respective derivative scaled by a parameter that depends on trial specific latency. Both parameters are scaled by a trial specific amplitude parameter (Figure 2). This model is easily extended to allow each trial to be described by multiple waveforms (each representing an underlying signal). We will first treat the single waveform case and then extend it to multiple waveforms.

### Model

The EEG/MEG data are in the 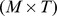 matrix 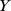 consisting of 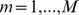 trials of length 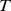. Each single-trial 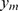 can now be modeled as a waveform plus its derivative:
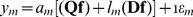
(1)


In Eq. 1 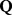 is a 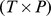 matrix containing the 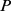 basis functions, 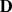 is a 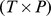 matrix containing the first-order derivatives of the basis functions, and 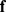 is a 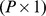 vector containing the 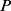 coefficients of the waveform. 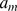 is the trial specific amplitude parameter and 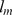 is the trial specific latency parameter. 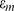 is the noise term distributed as 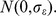


In order to model all trials at once we rewrite Eq. 1. We first move 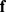 outside the brackets and replace 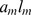 with 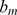. The model then becomes:
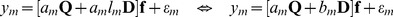
(2)


Then, by using the 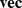 operator (stacking the columns of a matrix), the model for all 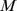 trials can be rewritten as:
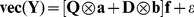
(3)

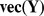
 contains the stacked data 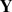. 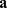 is an 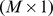 vector containing the single-trial amplitude parameters, 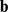 is an 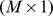 vector containing the single-trial latency parameters. 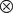 denotes the Kronecker Product.

In this model the type and number of (orthogonal) basis functions must be set a priori (matrix 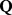). Note that a sufficient number of basis functions can be determined via model selection (see Model selection). In general any set of flexible basis functions can be used to model the waveform. In the current implementation we use a set of orthogonal polynomial basis functions since they are flexible enough to describe the waveforms. Also, polynomial basis functions are easy to compute and their derivatives can be obtained analytically. By default the number of basis functions is set to 20.

### Parameter Estimation

The SWALE model thus estimates both the waveform and trial specific amplitude and latency parameters from the data. Parameter estimation is split in two parts that are applied iteratively until convergence: estimation of the waveform 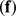 and estimation of single-trial amplitude 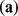 and latency 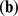 parameters. Each part has a linear solution and thus can be solved easily. For the estimation of the waveform the least squares estimator is:
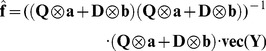
(4)


For estimation of single-trial amplitude and latency the least squares estimator is given by:
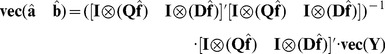
(5)where 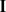 is the 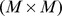 identity matrix.

**Figure 3:**
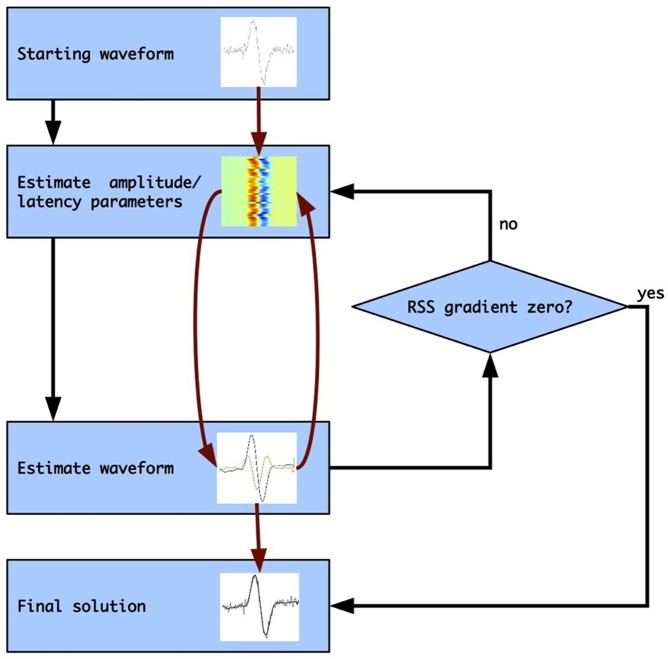
Parameter estimation procedure.

The estimation procedure thus consists of two parts that can be solved linearly. Applying these two parts iteratively leads to convergence of the overall solution (cf. [14]). The detailed procedure is as follows (see Figure 3): First, the grand average of the data is used as starting waveform for the iteration procedure. For this waveform the amplitude and latency parameters are estimated using Eq. 5. This set of amplitude and latency parameters is thereafter used to re-estimate the waveform using Eq. 4. This waveform will be slightly different from the starting waveform (but closer to the actual waveform). Subsequently, the amplitude and latency parameters for this updated waveform are estimated using Eq. 5. These steps (calculating amplitude/latency parameters and the waveform) are repeated until the decrease in Residual Sums-of-Squares (RSS) is negligible. Note that the starting waveform does not need to have any relation with the estimated waveform.

**Figure 4:**
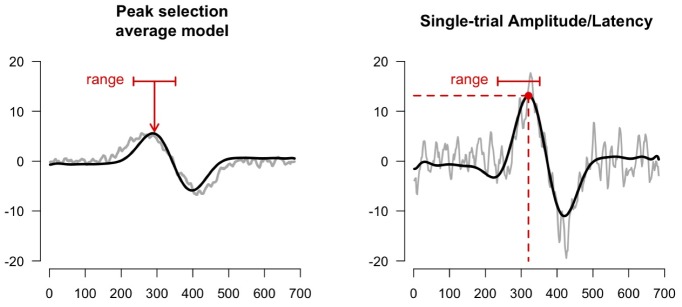
Single-trial detection. First a peak of interest must be identified in the averaged model by specifying a range (left panel, red line). Second, this range is used to estimate maxima/minima at each single-trial (right panel, red point).

### Estimating Single-trial Amplitude and Latency

After convergence of the estimation procedure single-trial estimates for a specific peak of interest can be obtained. In order to do so one must first identify the peak of interest in the average model (by specifying a range). For example, in Figure 4 (left panel) the positive deflection at 300 ms is selected as the peak of interest.

To obtain amplitude and latency estimates for this peak, at each modeled single-trial (Eq. 3) the maximum/minimum deflection within the range is identified (Figure 4, right panel, red point). The time-point of this maximum/minimum is the latency of the single-trial, the value at this latency is taken as the amplitude of the single-trial. This procedure can also be followed for models with multiple waveforms (see Multiple signals). The estimation is then performed for each waveform separately.

**Figure 5:**
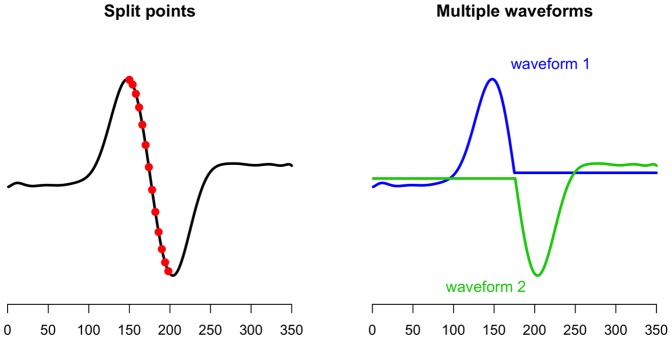
Procedure for splitting the waveform in multiple parts. Left panel shows the average model for one waveform. This average waveform is split into two parts using all time-points (left panel, red points) between consecutive peaks. The split that leads to the best fitting model is taken as the optimal model (right panel).

**Figure 6:**
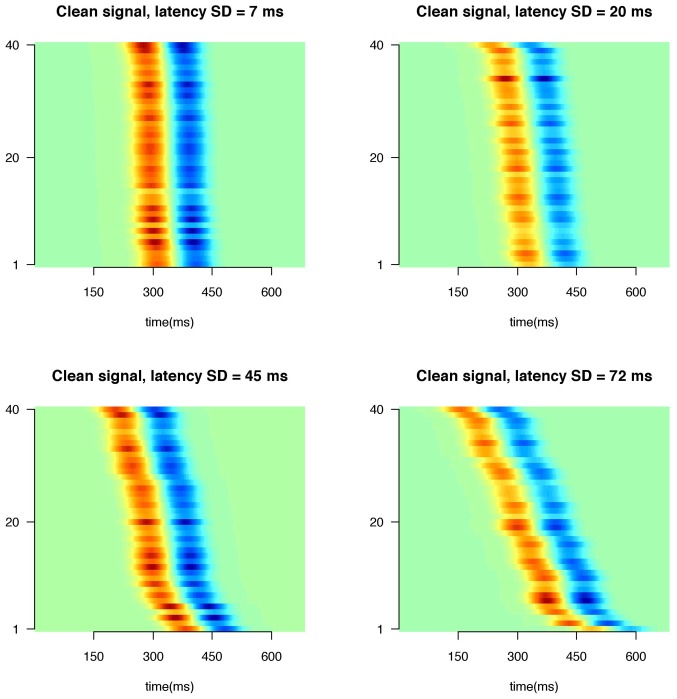
Rasterplots of actual signal using different amounts of latency variation of the signal. X-axis indicates time, y-axis indicates trial number, colors indicate signal amplitude.

**Figure 7:**
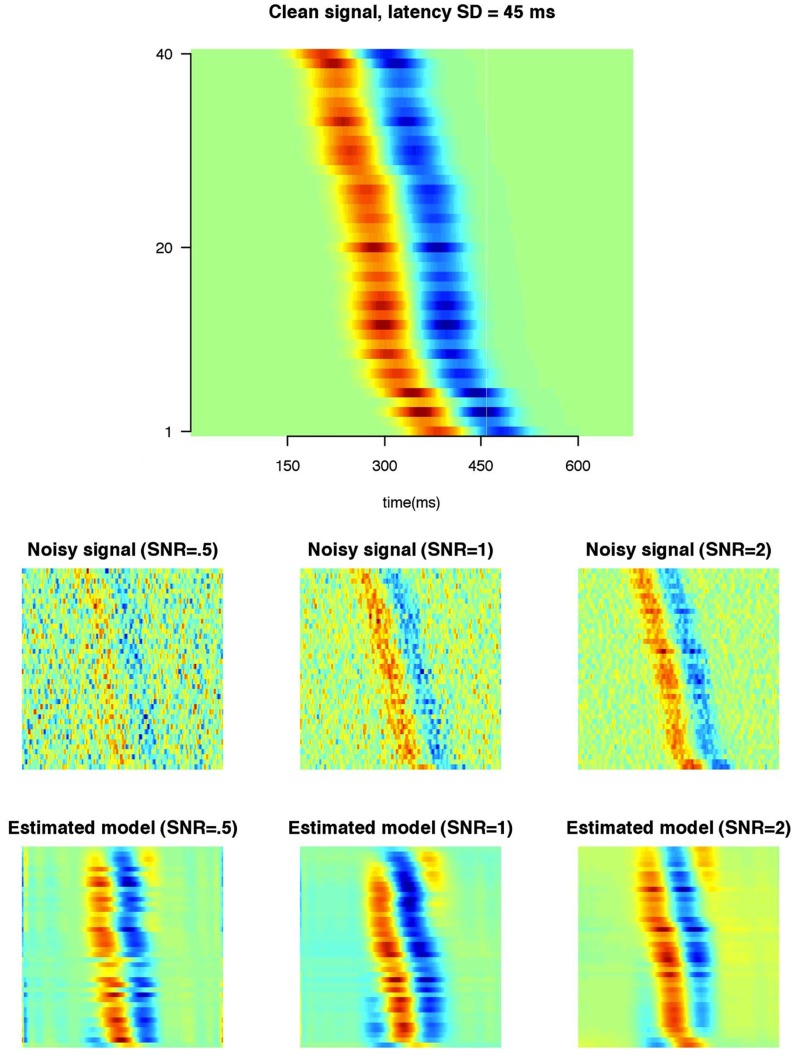
Rasterplots of actual signal, noisy signals and estimated models under different levels of SNR. X-axis indicates time, y-axis indicates trial number, colors indicate signal amplitude.

### Multiple Signals

In the method explained above, it is assumed that each EEG/MEG trial can be modeled by one waveform. This implies that, at each trial, the entire waveform is affected by one latency and amplitude parameter. This may not be a plausible model from a physiological point of view, since each signal may be characterized by signal specific amplitude and latency parameters. Therefore, the method should be extended to model multiple underlying signals, each with a separate waveform. SWALE uses a model selection approach to determine whether the signal can best be modeled by one or multiple waveforms.

**Figure 8:**
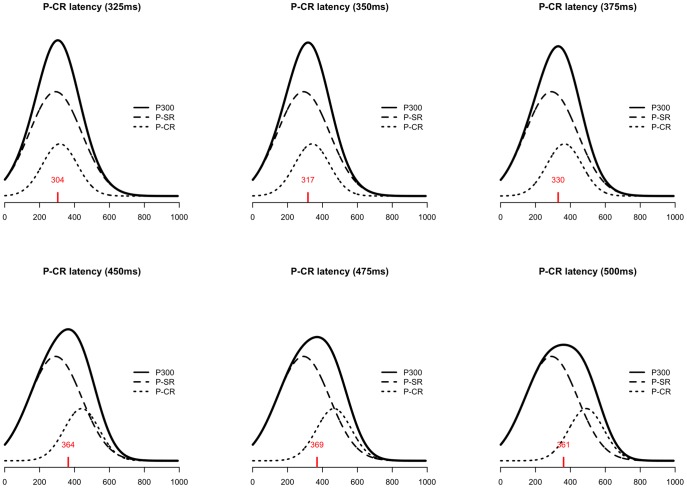
Simulated data showing effect of late P-CR latency on P300 latency. Dashed line indicated the early P-SR, dotted line indicated late P-CR. Solid line indicates the combined waveform of early P-SR plus late P-CR, that is, the P300. Vertical red line and number indicates the latency of the combined waveform (P300).

#### Selecting multiple waveforms

For the selection of multiple waveforms a time-range of interest must be specified. This may be data-driven of theory-driven. Once peaks within this range are specified, we proceed in the following manner. First, a model with one waveform is fitted (see Parameter estimation). The estimated waveform from this analysis is then split into two waveforms (Figure 5, left panel, red points). The waveform is split at each time-point between two peaks within the range of interest. For each split the amplitude and latency parameters are estimated using Eq. 5 and the model fit is calculated. Figure 5 (right panel) shows an example of a waveform split. In this example the waveform is split into two parts, rendering two waveforms for which amplitude and latency estimates are calculated. From all the calculated splits between two peaks we choose the split that has the best fit.

**Figure 9:**
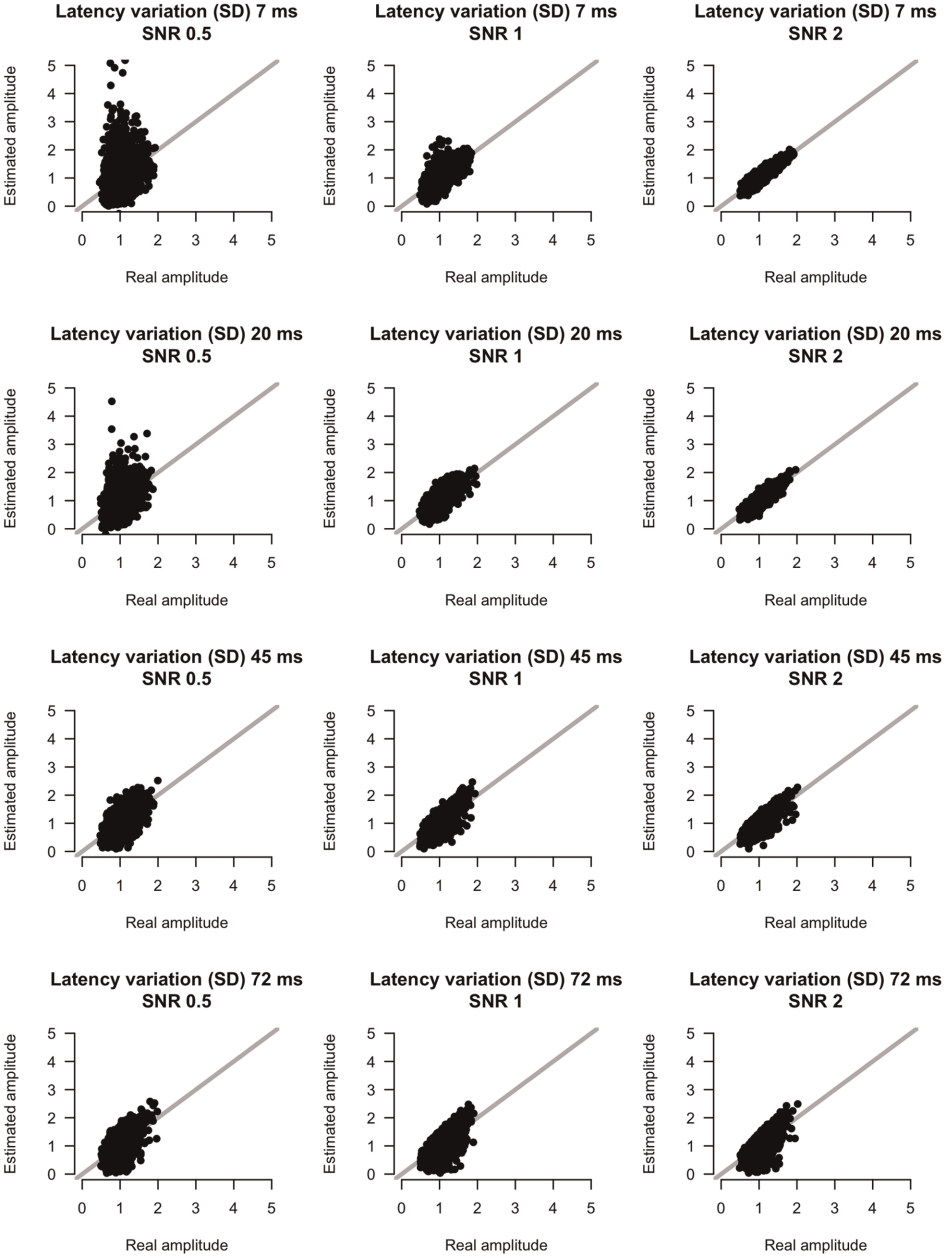
Real versus estimated amplitude estimates for different amounts of SNR and latency variation. X-axis indicates real amplitude, y-axis indicates estimated amplitude.

**Figure 10:**
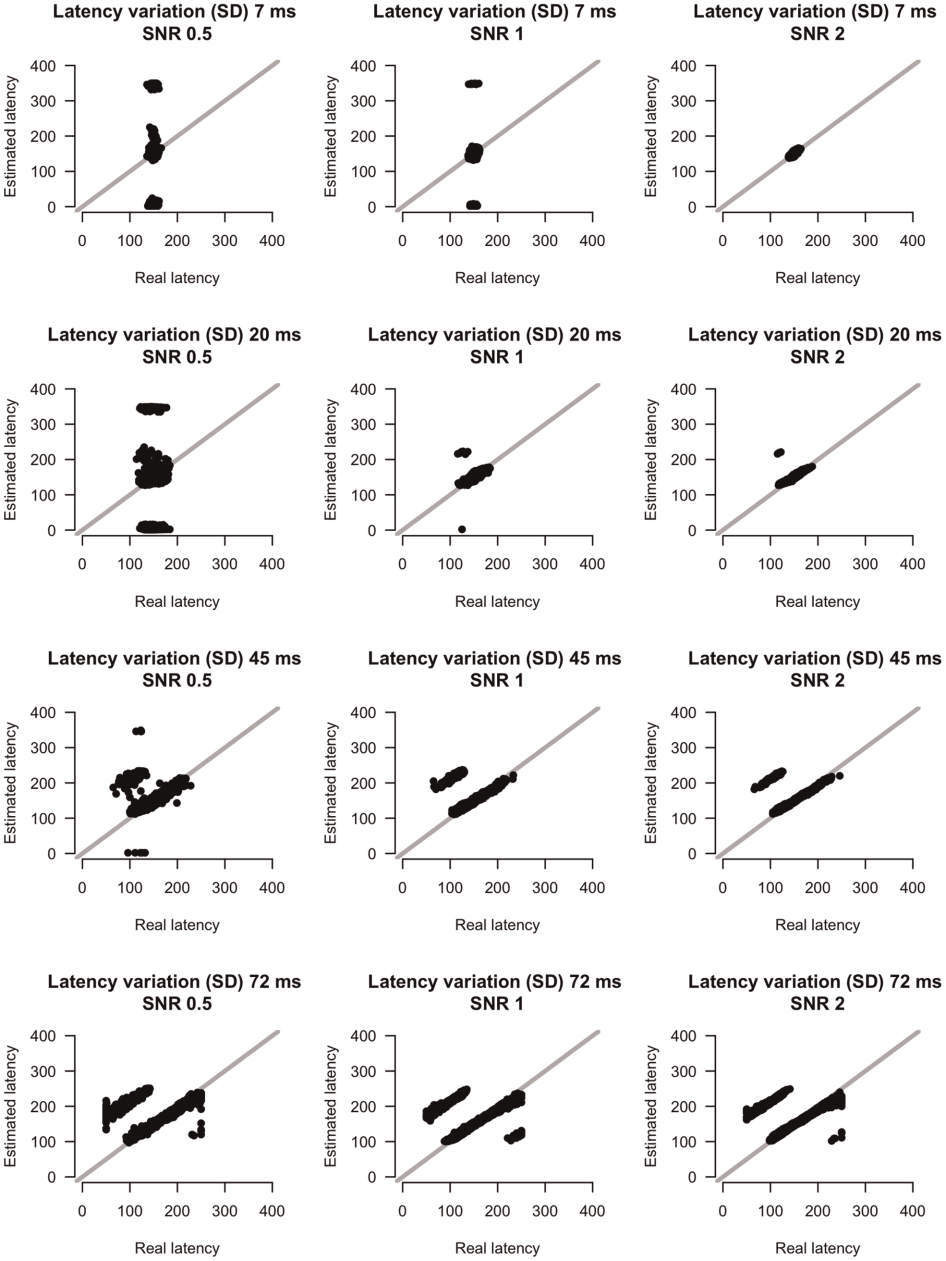
Real versus estimated latency estimates for different amounts of SNR and latency variation. X-axis indicates real latency, y-axis indicates estimated latency.

### Model Selection

It is necessary to decide how many basis functions best describe the waveform and to decide if multiple waveforms are required to describe the data. Increasing the number of basis functions, or increasing the number of waveforms, leads to increased model fit, but also to increased model complexity. A sparse model with good fit is preferred. When comparing models it is therefore necessary to take into account both model fit and model complexity (i.e. the number of parameters in the model). A good method to accomplish this is by using the AIC [15]. The AIC is calculated as follows:
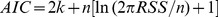
(6)


In Eq. 6, 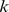 denotes the number of parameters (in this case 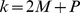) and 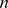 denotes the number of data points (in this case 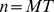). The model with the smallest AIC value is taken as the best model.

We can now determine the optimal number of basis functions and the optimal number of waveforms to describe the data. The optimal number of basis functions can be determined by analyzing the data using different numbers of basis functions at each run. To decide how many waveforms best describe the data, models with splits at different points, and differing numbers of splits can be compared with the AIC, using the procedure explained in the previous section.

### Simulations

To assess bias in parameter estimates and the model selection procedure we performed simulations using synthetic data. We simulated 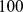 datasets each consisting of 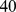 trials with 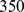 samples (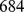
ms). At each trial we simulated two signals, one with a peak at 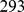 ms and one with a peak at 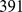 ms. Each peak varied over trials in amplitude (lognormal distributed with mean 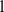 and sd 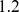, restricted between 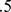 and 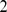), and latency (normally distributed with mean 0 and sd 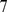, 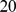, 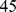, or 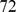 ms). Values of the distribution of amplitude and latency parameters were derived from [9], [10]. Figure 6 shows the signal using different amounts of latency variation. We simulated two conditions. In the ‘fixed’ condition the amplitude and latency parameters were the same for both peaks, that is, within a trial the peaks were shifted and scaled by the same amount. In the ‘free’ condition both amplitude and latency parameters varied independently between peaks, that is, within a trial each peak was shifted and scaled by different values, thus simulating two separate signals. Assuming total independence between amplitudes and latencies of different signals is physiologically not very plausible. For simulation purposes however it reflects a ‘worst case scenario’ as peaks might cancel each other out or have reversed polarity. All simulations were performed using 20 basis functions for the estimated waveform under three signal-to-noise (SNR) conditions (SNR  = 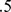, 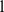, and 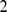), using correlated noise. Noise was simulated using an AR(5) process with coefficients estimated from baseline trials of the empirical data (see Empirical application). The SNR values are commonly found in EEG/MEG studies [2], [16]. Figure 7 shows an example of the signal, noisy data and estimated model under different SNR values. For the simulations assessing bias, the peak of interest was selected to be the positive peak at 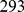
ms in the ‘fixed’ condition (range 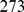 to 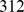
ms).

**Figure 11:**
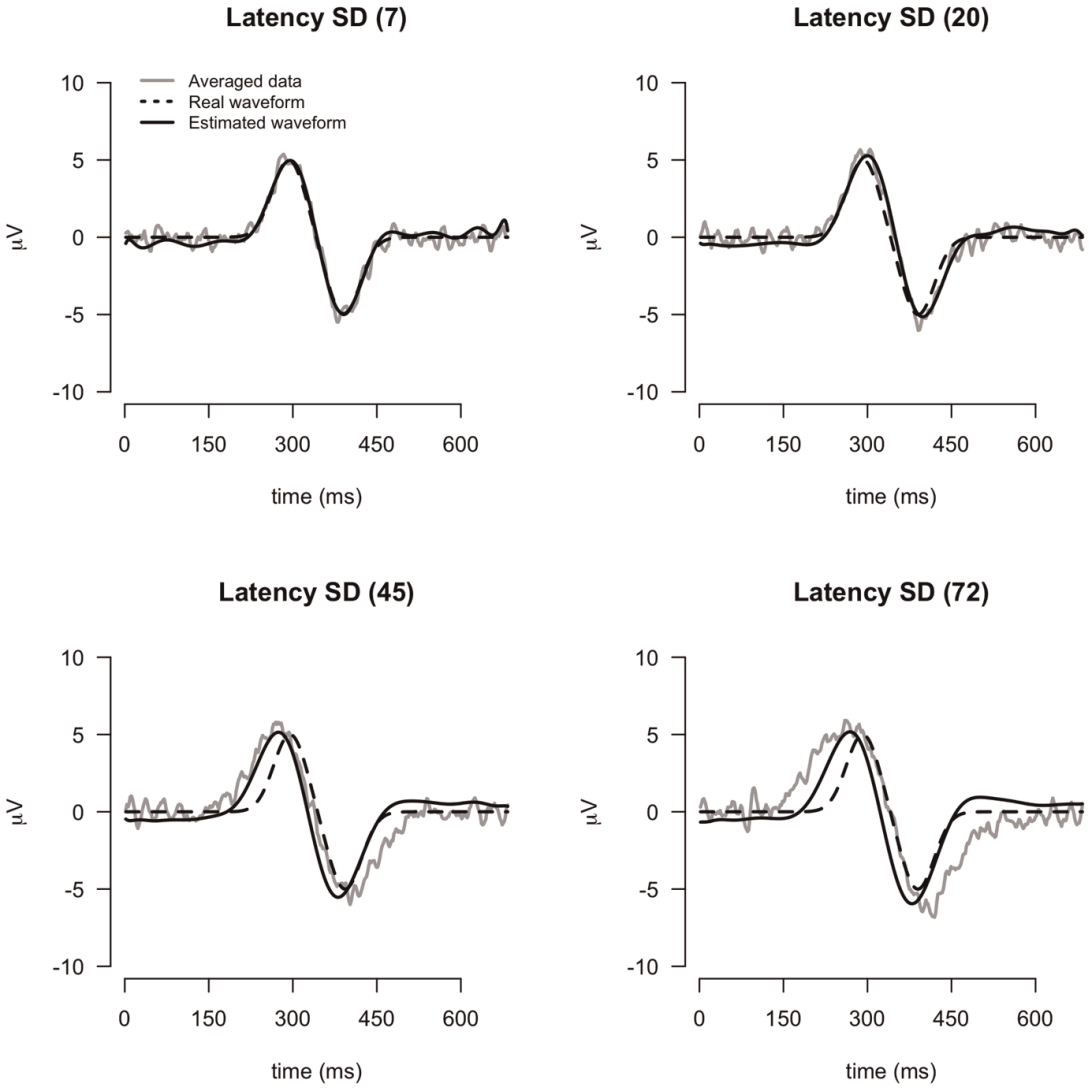
Relation of estimated waveform with increased latency variation.

### Empirical Application

To illustrate performance we applied the SWALE method to single-trial EEG data from a choice reaction time (CRT) study. We analyzed the (pre-processed, artifact removed, detrended) stimulus-locked correct trials at the Pz electrode from a single subject. The dataset consisted of 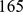 trials of 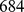
ms (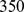 samples at 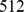
Hz). Performance was assessed in three ways. First, the solution of the SWALE method was checked to see if it correctly modeled the data. Second, the method was compared to standard peak-picking. Third, a functional test of the method was performed to see whether the SWALE method was able to delineate different processes assumed to underlie performance in CRT studies.

**Figure 12:**
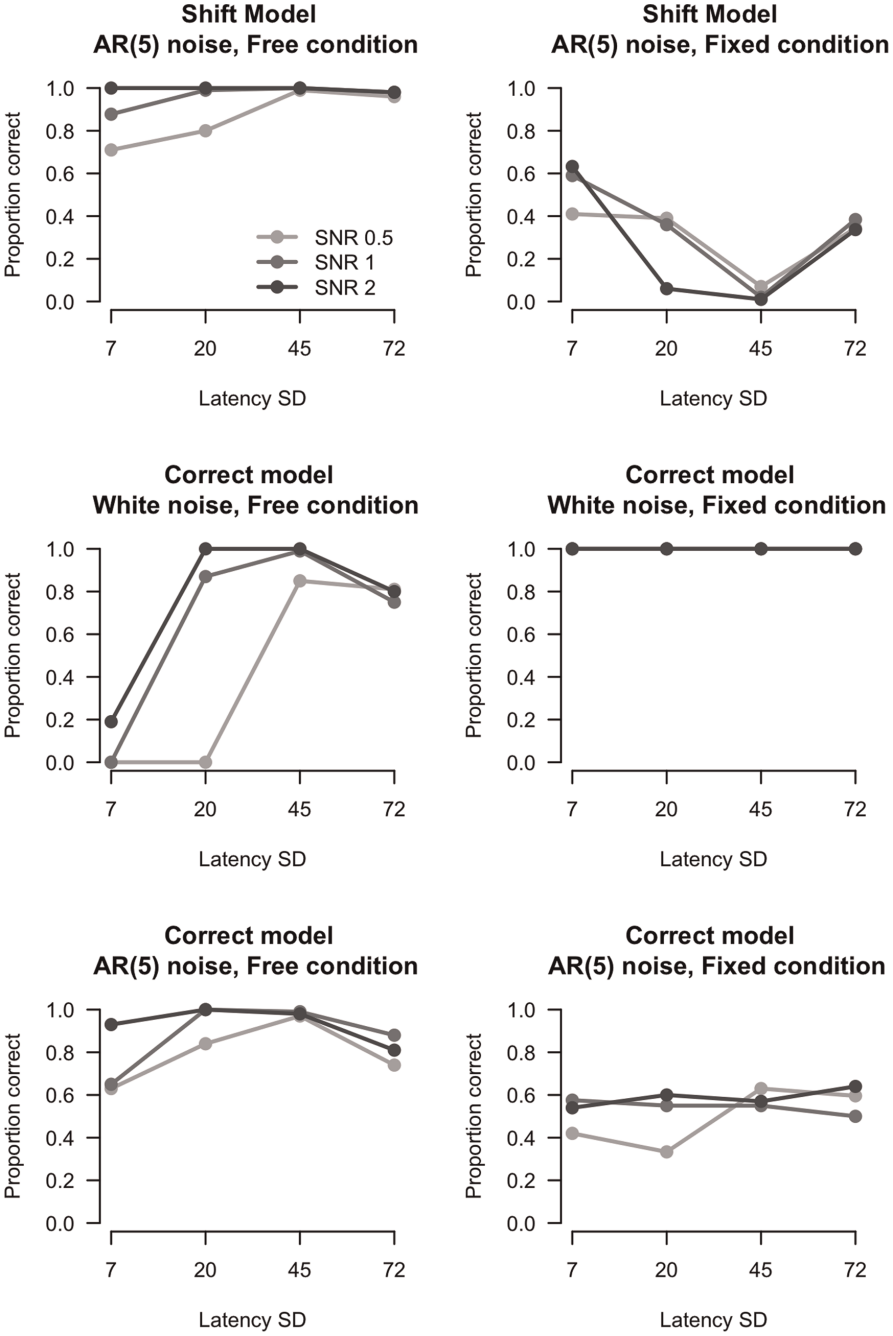
AIC model selection. X-axis indicates amount of latency variation. Y-axis indicates proportion correct model selections (in the ‘fixed’ condition a model with one waveform should be selected, in the ‘free’ condition a model with two waveforms should be selected).

Choice reaction time studies usually elicit a late positive component termed the P300. In choice reaction time studies the elicited P300 is also known as the P3b. This to make a distinction with the P3a which is elicited when viewing novel stimuli in, for example, an oddball paradigm. We will use the term P300 to indicate the P3b. The P300 component peaks at around 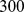
ms after stimulus presentation, is often found to be correlated with reaction time [17] and is usually linked to stimulus evaluation processes. There is evidence that the P300 consists of two components, one reflecting stimulus evaluation (early P-SR) and one reflecting response selection (late P-CR) [18], [19]. The early P-SR peaks earlier in time than the late P-CR and is unrelated to choice RT (it peaks at approximately the same time in every trial), the late P-CR peaks later and its latency is related to choice RT (that is, RT depends on the latency of this peak). In easy choice RT tasks the early P-SR and late P-CR overlap in time. With increasing difficulty the late P-CR will peak later than the early P-SR, thereby prolonging the latency of the P300 peak. Although these differences were mainly shown by manipulating task complexity [18], the effect is also shown within single tasks [17] by contrasting the waveforms of fast and slow reaction times. Figure 8 (adapted from [19], Fig. 5, p. 151) shows synthetic data reflecting these processes.

**Figure 13:**
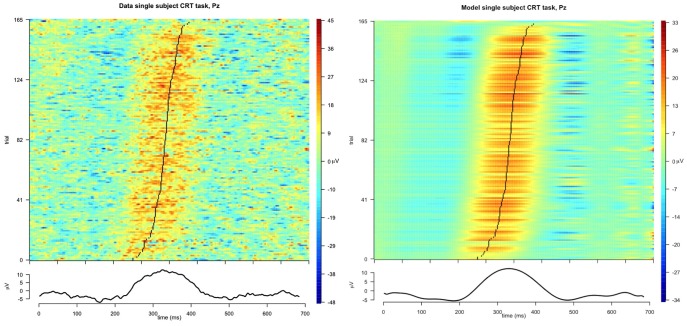
ERP plots of the single-trial data (left) and model (right). Trials are ordered by estimated latency of the first peak. Bottom panels show the averaged ERPs. X-axis indicates time, y-axis indicates trial number.

**Figure 14:**
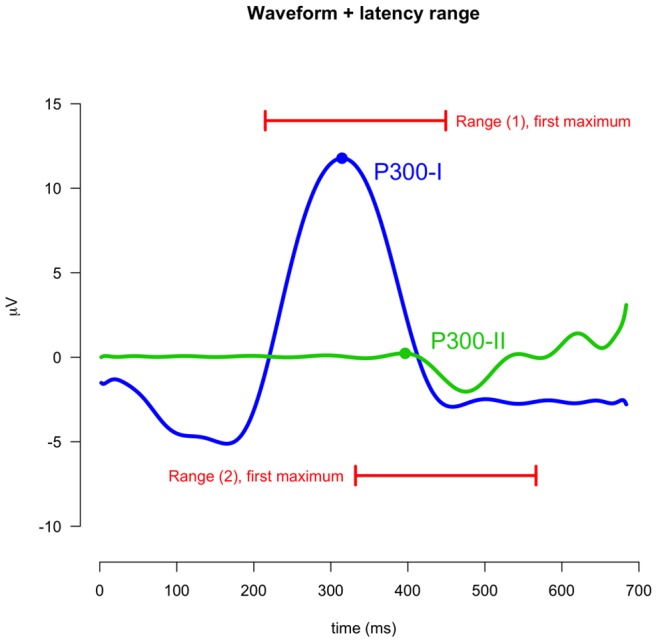
Waveforms for the optimal model (P300-I and P300-II) with the specified range used to select the peaks.

The upper panels show the effect of increasing delay of the P-CR on the latency of the P300 when responses within a task are relatively fast (that is, when the P-CR appears early). In this case both early P-SR and late P-CR overlap and, as can be seen from the latency of the P300 (Fig. 8, upper panels, red numbers), increases in late P-CR latency lead to approximately the same increase in P300 latency. The bottom panels of Figure 8 show the effect of increasing latency of the P-CR on P300 when the P-CR is slow. As can be seen, the resulting P300 is lower in amplitude and wider, and the latency of the P300 (Fig. 8, lower panels, red numbers) does not increase constantly with late P-CR latency (it even decreases when late P-CR latency is very long). The SWALE model should be able to detect these different processes, and SWALE derived amplitude and latency estimates of slow and fast trials should be consistent with this view. In other words (i) model selection should indicate a model with multiple waveforms, (ii) RT should correlate with P300 latency in fast trials, but not in slow trials, and (iii) RT should correlate with P300 amplitude in the slow trials but not in fast trials.

**Figure 15:**
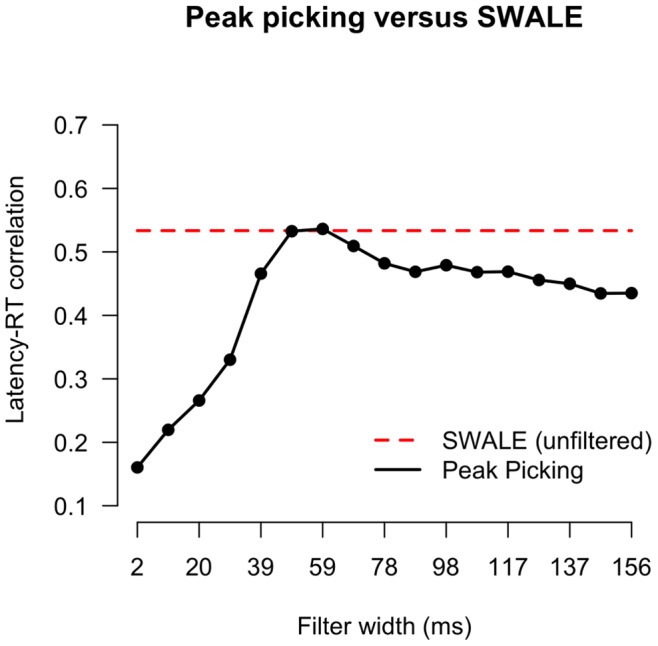
Correlation of estimated latency and RT for Peak-picking (black line) using different filter widths (x-axis) versus SWALE (red line).

#### Methods

The SWALE method was applied using default settings (20 basis functions). To assess multiple signals, the model with one waveform was compared to a model consisting of two waveforms, modeling a positive (P300) peak, and a peak preceding or following the positive (P300) peak. To further assess the performance of the SWALE method we compared SWALE with standard peak-picking. For the peak-picking method we smoothed the data with a Gaussian smoothing kernel where the width was varied form 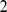 to 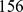
ms. We selected, for each trial, the maximum value within a window (between 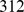 and 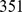
ms). For the functional application amplitude and latency estimates of each waveform were correlated with RT of the fastest responses (RTs within the first quartile, 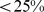) and slowest responses (RTs within the fourth quartile, 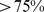) separately.

## Results

### Simulations

Figures 9 and 10 show scatterplots of amplitude and latency estimates versus real amplitude and latency values for four levels of latency variation and four SNR levels. Amplitude estimates improve with increasing SNR. Latency variation has a less pronounced effect on amplitude estimates, except in the lower SNR condition. In this condition, low latency variation gives rise to inaccurate amplitude estimates. For the latency parameters the pattern is qualitatively the same. Overall, the method thus estimates amplitude and latency parameters accurately. Only if SNR is low and latency variation is low, estimates are unreliable.

**Figure 16:**
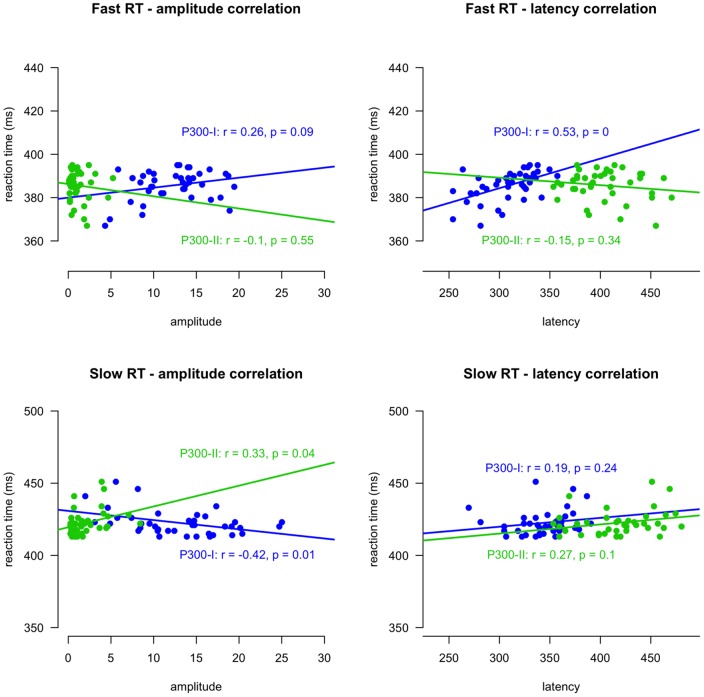
Correlations with RT, amplitude and latency for fast and slow trials.

#### Waveform estimation

The SWALE procedure also estimates the waveform from the data. Note that if the waveform is estimated from the averaged data, this waveform will be affected by the amount of latency variation. SWALE however should produce estimates that are independent of latency variation. To check this, we compared the true waveform to the waveform estimated from the averaged data and the waveform estimated by SWALE, under different levels of latency variation (cf. Figure 11). As can be seen, the SWALE waveform is closer to the actual (true) waveform than is the waveform based on the averaged data. With increased latency variation, the averaged waveform is wider in shape, while the SWALE waveform is relatively unaffected.

#### Model selection

Figure 12 shows the proportion correct model selections in the ‘fixed’ and ‘free’ condition for four levels of latency variation and three SNR levels (top panels). In the ‘free’ condition the correct model (two waveforms) was selected in almost all of the datasets and performance was relatively unaffected by SNR. In the ‘fixed’ condition the correct model was selected much less often, the procedure indicated too often that two (instead of one) waveforms were required. This pattern of results can have two underlying causes. First, performance of the AIC might be degraded due to the incorrect model (i.e. the model to estimate the waveform is *not* the model used to simulate the data). Second, the AIC might be affected by correlated noise. To test these assumptions we simulated data with the correct model (i.e. the SWALE model (Eq. 1) was used to simulate the data) under conditions of uncorrelated (white) and correlated noise. Figure 12 (middle and lower panels) shows the results from these simulations. Results from the correct model with white noise (middle plots) indicate optimal performance. In the ‘fixed’ condition (middle plot, right panel), it can be seen that model selection is perfect for all levels of latency variation and SNR. For the ‘free’ condition (middle plot, left panel) model selection is accurate with higher levels of latency variation and is better under higher SNRs. This is expected as models with hardly any latency variation can be modeled accurately with a model with one waveform. The effect of correlated (AR(5)) noise on the AIC can be clearly seen when comparing the middle plots with the bottom plots. Accuracy drops for the fixed condition, and increases for the free condition, indicating that correlated noise makes the AIC prefer the more complex model.

### Empirical Application

#### Model fit and model selection

Model selection indicated that a model with two waveforms (with the peak following the positive P300 peak) provides the best description of the data. Figure 13 shows ERP plots of the single-trial data (left) and model (right). Trials are ordered by estimated latency of the first peak, the bottom panels show the averaged ERPs. As can be seen in Figure 13 the model describes the single-trial data very well. Figure 14 shows both waveforms for the optimal model. As can be seen, the first peak corresponds to the P300 complex (containing both early P-SR and late P-CR), while the second peak corresponds to the part of the late P-CR not overlapping with the early P-SR. We will term these waveforms the P300-I and P300-II respectively.

#### Comparison with peak-picking

Figure 15 shows the correlations between P300-I peak latency and RT both for SWALE and peak-picking. Note that for peak-picking the filter width was varied and that the SWALE estimate is constant (dashed red line) as it was only calculated on the unsmoothed data. Results show that, only given the optimal filter width (black line), peak-picking and SWALE results are the same.

#### Functional assessment

Trials were divided into fast trials (all RTs within the first quartile; 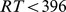
ms) and slow trials (all RTs within the fourth quartile; 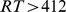
ms), leaving 41 trials in each condition. Figure 16 shows the correlations of amplitude and latency of P300-I and P300-II peaks with fast and slow RT. For fast RTs the amplitude parameters (upper left panel) of neither peak correlated significantly with RT, although there was a trend (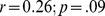) for the amplitude of the P300-I to correlate positively with RT. Analysis of latency parameters of fast responses (upper right panel) shows a positive correlation between latency of the P300-I (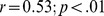) and RT but no correlation between latency of the P300-II and RT. This is consistent with the P-SR and P-CR overlapping for fast RTs, with P-CR influencing the latency of the entire P300 (modeled by the P300-I). For slow RTs the amplitude parameters correlated both significantly with RT. Amplitude of the P300-I correlated negatively with RT (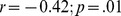), amplitude of the P300-II correlated positively with RT (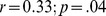). Latency parameters of neither peaks correlated with slow RT, although there was a trend for the latency of the P300-II to correlate positively with RT (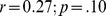). These results are consistent with the view that for slower RTs P-SR and P-CR overlap to lesser extent due to the longer latency of the P-CR: For slower RTs amplitude parameters of the first peak (P300-I containing P-SR and P-CR) are lower. Furthermore, for longer RTs the amplitude of the non-overlapping part of the P-CR (P300-II modeled by the second peak) are higher and there is a trend that the the latency of this peak is longer.

## Discussion

The SWALE framework can reliably estimate single-trial waveform, amplitude and latency parameters in data containing multiple signals. Simulations have shown that estimates of amplitude and latency are within acceptable limits. Only if SNR is low and latency variation is low, estimates become unreliable. In testing for the absence/presence of multiple signals the AIC has a preference for more complex models, mainly due to correlated noise in the data. An application to a CRT study has shown that without specifying an a-priori template, the model fits the data well and that estimates of waveform, amplitude and latency produce sensible results. Also, the estimated multiple waveforms were consistent with a P-SR/P-CR model often used in CRT studies.

There are several extensions that can be made to our model. First, accuracy of model selection will improve, if the data are prewhitened by including a model for the temporal noise correlations. For example, de Munck et al. [14] account for temporal correlations in the data. Including similar methods might improve performance. Second, we used polynomial basis functions. It may be worthwhile to generalize the method to localized wavelet functions, to obtain a more compact localized representation of the waveforms. For example, Quian Quiroga [20] and Wang et al. [21] have shown considerable advantages with using wavelets to estimate waveforms of single-trial EEG data. Third, extensions can be made regarding the determination of multiple underlying signals. Currently, these waveforms are obtained in a data-driven way. This even works for signals that are (partially) overlapping in time, as long as these signals do not overlap in the same manner in each single-trial (i.e. if signals are perfectly correlated, in amplitude and latency, they can be modeled with one waveform). A possible solution to this drawback is to include information from multiple electrodes, allowing the method to better distinguish overlapping signals. When prior information on underlying signals (e.g. from theory) is available, this can easily be incorporated in the framework. By using a-priori defined waveforms directly (i.e. not estimating them), hypotheses regarding these signals can be tested using model selection. This extends current applications with the ability to explicitly test whether these waveforms give a good description of the data. The SWALE framework therefore provides a flexible framework for the estimation of single-trial EEG/MEG data.
